# Ranking factors involved in diabetes remission after bariatric surgery using machine-learning integrating clinical and genomic biomarkers

**DOI:** 10.1038/npjgenmed.2016.35

**Published:** 2016-10-26

**Authors:** Helle Krogh Pedersen, Valborg Gudmundsdottir, Mette Krogh Pedersen, Caroline Brorsson, Søren Brunak, Ramneek Gupta

**Affiliations:** 1 Department of Bio and Health Informatics, Technical University of Denmark, Kongens Lyngby, Denmark; 2 Department of Disease Systems Biology, Novo Nordisk Foundation Center for Protein Research, Faculty of Health and Medical Sciences, University of Copenhagen, Copenhagen, Denmark

## Abstract

As weight-loss surgery is an effective treatment for the glycaemic control of type 2 diabetes in obese patients, yet not all patients benefit, it is valuable to find predictive factors for this diabetic remission. This will help elucidating possible mechanistic insights and form the basis for prioritising obese patients with dysregulated diabetes for surgery where diabetes remission is of interest. In this study, we combine both clinical and genomic factors using heuristic methods, informed by prior biological knowledge in order to rank factors that would have a role in predicting diabetes remission, and indeed in identifying patients who may have low likelihood in responding to bariatric surgery for improved glycaemic control. Genetic variants from the Illumina CardioMetaboChip were prioritised through single-association tests and then seeded a larger selection from protein–protein interaction networks. Artificial neural networks allowing nonlinear correlations were trained to discriminate patients with and without surgery-induced diabetes remission, and the importance of each clinical and genetic parameter was evaluated. The approach highlighted insulin treatment, baseline HbA1c levels, use of insulin-sensitising agents and baseline serum insulin levels, as the most informative variables with a decent internal validation performance (74% accuracy, area under the curve (AUC) 0.81). Adding information for the eight top-ranked single nucleotide polymorphisms (SNPs) significantly boosted classification performance to 84% accuracy (AUC 0.92). The eight SNPs mapped to eight genes — *ABCA1, ARHGEF12, CTNNBL1, GLI3, PROK2, RYBP, SMUG1* and *STXBP5* — three of which are known to have a role in insulin secretion, insulin sensitivity or obesity, but have not been indicated for diabetes remission after bariatric surgery before.

## Introduction

Type 2 diabetes mellitus patients are increasingly recognised to experience improved glycaemic control following bariatric surgery,^1^ and a growing number of randomised control trials consistently report surgery to be more effective for controlling obese Type 2 diabetes patients than various medical/lifestyle interventions.^2^ Furthermore, obese type 2 diabetes patients who have undergone bariatric surgery present with fewer complications compared with surgery-naive patients.^3^ Consequently, new guidelines from the second Diabetes Surgery Summit recommend the use of bariatric surgery as an antidiabetic treatment for Type 2 diabetes patients with body mass index (BMI) ⩾40 kg/m^2^ or BMI 35.0–39.9 kg/m^2^ suffering from inadequately controlled hyperglycaemia, and further suggest considering surgery for patients with BMI 30.0–34.9 kg/m^2^ and inadequately controlled hyperglycaemia.^2^ Several mechanisms seem to contribute to surgery-induced diabetes remission, including gut hormone and microbiota changes, bile acid reabsorption and caloric restrictions.^4–7^ In an effort to curb the epidemic of obesity and diabetes, a growing number of people are turning to gastric bypass surgery. However, not all patients achieve surgery-induced diabetes remission, and the remission rate depends on surgery procedure,^8^ clinical presentation, patient risk factors^9,10^ and patient genetic predisposition.^11^ Genome-wide association studies (GWAS) are attempting to uncover genetics that predispose an individual to good prognosis of diabetes remission (database of Genotypes and Phenotypes (dbGaP) accession number: phs000380.v1.p1) but not much has yet been published. The heritability of diabetes remission following bariatric surgery is largely unknown, but surgery-induced excess body weight loss has been found to be significantly more similar between first-degree relatives compared with unrelated individuals, including unrelated individuals living together,^12^ suggesting the involvement of a genetic component. However, despite increased focus in this area, the precise underlying molecular mechanisms and prognostic factors of remission remain incompletely understood. Such insight would improve selection of patients for bariatric surgery, and might hint at new pharmaceutically relevant biomarkers and targets. Consequently, it is of interest to investigate and identify phenotypic and genomic factors associated with surgery-induced diabetes remission. It is also of interest to identify patients unlikely to benefit in their diabetic condition to possibly avoid surgical risks where the diabetic condition is a major objective of the surgery, since the surgery procedure is not without risk, although the mortality rate and complication frequency are within reasonable range for elective surgery. Still, up to 15% of patients experience minor complications and 2–6% suffer from major complications with 2.5% and 5.1% requiring early reoperation or readmission after laparoscopic Roux-en-Y gastric bypass.^2^

The multifactorial genetic architecture of a complex disease like diabetes presents challenges in correlating genomic variation with phenotypic differences. Most existing methods for GWAS are single-locus/single nucleotide polymorphism (SNP) association-based approaches.^13,14^ Such methods are not able to capture correlations between SNPs and the burden of correcting for multiple-hypothesis testing necessitates ever increasing sample sizes. Furthermore, not many studies examine the interactions between genetic and clinical or environmental factors in part due to a substantially larger multiple-hypothesis-testing correction necessity for exhaustive combinatorial searches. Consequently, integrative network- and machine-learning-based approaches are gaining interest in the search for the missing heritability of many complex traits,^15^ with the promise of being able to harness information across SNPs as well as other data types.

**Figure 1:**
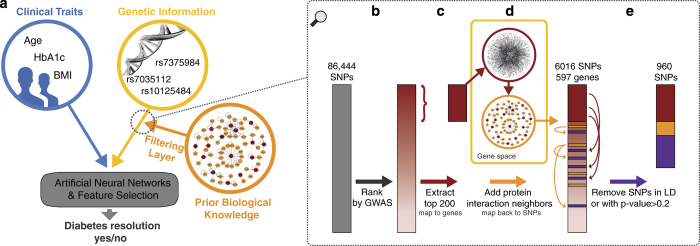
(**a**) General framework for integrating heterogeneous data types for patient stratification. In this study we focus on the three data types: clinical traits, genetic information and protein–protein interactions. Panels (**b**–**e**) illustrate the approach for compiling an enriched subset of candidate SNPs by utilising prior biological knowledge. Essentially, the top 200 GWAS SNPs were expanded using protein–protein interaction data, see text for details.

Here we propose a methodology that allows for the combination of factors, originating from heterogeneous data types to investigate the effect of multiple variables simultaneously and uncover correlations between variables (Figure 1a). The data set in the present study of surgery-induced diabetes remission includes clinical traits and SNP data from the CardioMetabochip.

Testing all feature combinations from a 200,000 SNP CardioMetaboChip array is computationally infeasible (and a severe multiple-testing burden). To overcome this obstacle, we initially ranked single SNP associations using univariate tests adjusted for age and sex and prioritised a set of 200 markers, through a typical genome-wide association approach. We expanded the most promising associations with prior biological knowledge to generate a larger SNP set, likely encompassing a wider range of relevant biological signals. This larger SNP set, together with a set of clinical prognostic factors, were then feature-selected through machine-learning. This involved training artificial neural networks to discriminate between patients with and without surgery-induced diabetes remission; over half a million predictive models were built where their performance helped assess the importance of individual features used in the various models. In order to reduce patient similarity between training and test sets, samples with similar clinical properties were removed from the entire data set ahead of training the models.

The main goals of the study were to stratify individuals based on clinical and genomic factors that determine their diabetic response to surgery, and to eventually identify factors that have an important role in this response, several of which might be overlooked in individual feature association tests.

## Results

### Assessing informative clinical traits

Univariate analysis showed significant associations of multiple baseline characteristics with surgery-induced diabetes remission (Table 1). Younger age, lower baseline HbA1c and baseline serum glucose levels, higher baseline serum insulin levels, and use of biguanides, but not insulin or insulin-sensitising agents, were all associated with diabetes remission. In accordance with previous findings, this together suggests a higher likelihood of diabetes remission for less severe or progressed diabetes patients.^9,16–18^

**Figure 2:**
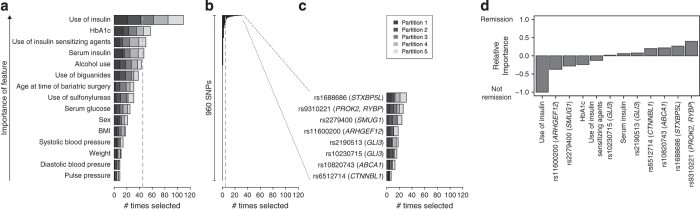
Ranking of features. (**a**–**c**) The number of times (out of 125) a given clinical feature (**a**) or SNP (**b** and **c**) was selected in the forward feature selection approach. The more times selected, the more important the given feature is in predicting diabetes remission. (**d**) Relative importance of input variables for diabetes remission, highlighting the directionality of the different features (positive values indicate that high values/minor alleles is associated with diabetes remission, whereas negative values indicate that high values/minor alleles/taking the medication is associated with failure of diabetes remission). The plot shows the average relative importance for the five outer cross-validation folds.

Multivariate feature ranking (through artificial neural network models) also highlighted factors associated with preoperative disease severity as important for discrimination between remitters and nonremitters (Figure 2a). Insulin therapy was selected in 109/125 feature selections, whereas use of insulin-sensitising agents, baseline serum insulin level and baseline HbA1c were selected in 50, 47 and 57 feature selections, respectively.

### Assessing informative genetic information

Single SNP association from the *ca.* 200,000 marker Cardiometabochip did not show any genome-wide significant associations, where the SNP with the best association score was rs2279400 (odds ratio=0.49, 95% confidence interval 0.36–0.66, *P* value=3.5×10^−6^; see Supplementary Figure S1). In the neural network multivariate models, eight SNPs (from eight separate genes) were selected at least once in each of the five outer cross-validation folds (Figures 2b,c) and listed in Table 2. These eight SNPs include the top GWAS SNP (rs2279400), but interestingly, also three SNPs with weaker *P* values originating from the protein interaction network expansion of the top 200 GWAS SNPs (Supplementary Figure S1). We further investigated association scores with obesity, type 2 diabetes and glucose-stimulated insulin secretion-related phenotypes in summary-level data from the GIANT, DIAGRAM and MAGIC consortiums. Of the 18 included traits and studies (described in Table 2), only one SNP showed associations with nominal *P* value<0.01 (rs11600200 in waist–hip ratio adjusted for BMI), indicating that the majority of the SNPs potentially point at either novel biological mechanisms underlying bariatric surgery-induced diabetes remission or low effect sizes not picked up in single-marker association studies. The eight SNPs were annotated to eight genes and, interestingly, three of these, *ARHGEF12*, *RYBP* and *STXBP5L*, overlap clusters of islet-selective (compared with five non-islet cell lines) open chromatin sites (altogether counting 1,512 genes).^25^ *STXBP5L* further has islet-selective open chromatin in the transcription start site or gene body,^25^ and its expression levels have been associated with HbA1c levels.^26^

In order to untangle the artificial neural network models to try to understand how the different clinical traits and SNPs coalesce in stratification of the patients, we investigated the relative importance and directionality of each variable within the models (Figure 2d). Use of insulin medication and high baseline HbA1c predisposes an individual to the non-remitter phenotype, whereas minor alleles for six of the eight SNPs are associated with higher likelihood of experiencing post-surgery diabetes remission.

### Performance of selected clinical traits and SNPs

**Figure 3:**
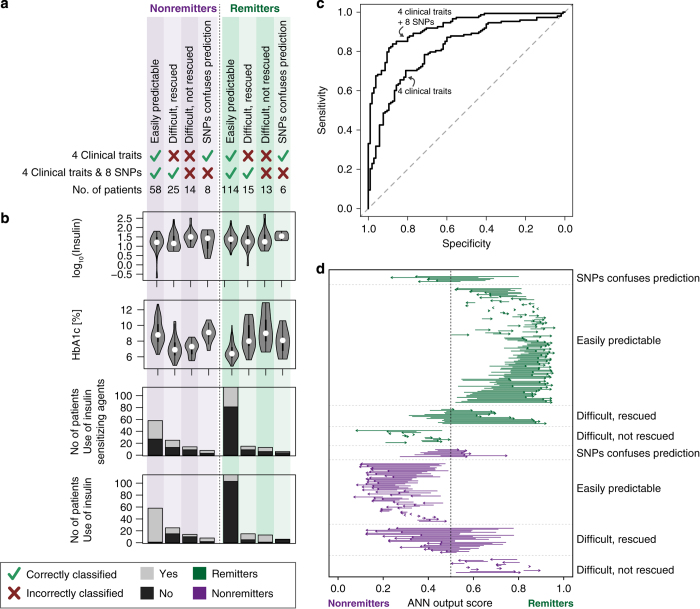
Patient breakdown. (**a**) The number of patients correctly or incorrectly classified in the internal validation step with an artificial neural network (ANN) predictor trained on the top four clinical traits alone, or the clinical traits+the eight top-ranked SNPs. (**b**) Distributions of variables for the eight different patient subgroups for the four top-ranked features. The violin plots in **b** indicate frequency distributions of the features (a kernel density plot), with the black bars indicating interquartile range and white circles the median value. (**c**) Receiver operating characteristic (ROC) curves for the two models: the four clinical traits alone or in combination with the eight SNPs. (**d**) Adding the SNPs pulls patients to the poles. The start of the arrows marks the output score from ANN trained on the four clinical traits, whereas the end (arrowhead) marks the output from ANN trained on both the four clinical traits and eight SNPs. During ANN training and evaluation, nonremitters are encoded as 0 and remitters as 1.

Internal cross-validation of the top-ranked four clinical traits (insulin treatment, baseline serum insulin levels, use of insulin-sensitising agents and baseline HbA1c levels) resulted in correct prediction of remission for 74% of the patients (area under the receiver operating characteristic curve (AUC)=0.81, Table 3). Adding information for the eight selected SNPs improved performance to an accuracy of 84% (AUC=0.92) and resulted in highly significant performance improvement as calculated by net reclassification improvement (NRI, NRI categorical: *P* value=1.45×10^−4^, NRI continuous: 2.81×10^−29^) and integrated discrimination improvement (*P* value=7.33×10^−25^; Supplementary Table S1), emphasising the potential of including these genomic markers (see also receiver operating characteristic in Figure 3c). Adding the eight SNPs further pulls the prediction output scores towards the extremes, thereby making the separation of remitters and nonremitters more distinct (Figure 3d). As a further validation, we tested the performance of eight random SNPs (drawn 1,000 times from the 960 tested SNPs, but excluding the selected eight SNPs) together with the clinical traits, which gave a performance similar to the clinical traits alone (Supplementary Table S2). Lastly, we verified that random data, simulated by permuting the labels, yielded (as expected) random performance for both models based on the clinical traits alone or in combination with the eight SNPs (Supplementary Table S2).

Although the four top-ranked clinical traits (insulin treatment, baseline serum insulin levels, use of insulin-sensitising agents and baseline HbA1c levels) independently explained variance in diabetes remission, no obvious cutoff could be applied to separate remitters from nonremitters (Figure 3a,b). Again, this emphasises the need for multivariate analysis to capture feature interactions in patient classification.

## Discussion

As big data approaches become more relevant in precision medicine,^27^ we demonstrate in this paper a follow-up to GWAS approaches and the ability to integrate clinical data as well as prior biological knowledge. We believe that such a paradigm can help identify subgroups of patients where genetic predisposition leads them to a different path, in response to surgery. For example, a group of 25 patients (9.9%) was incorrectly classified as remitters with the clinical traits alone, but correctly predicted to be nonremitters when including the eight most informative genomic markers (Figure 3a). The ability to identify this non-obvious patient group may rescue these individuals from undergoing an invasive surgery because of their genetic predisposition against diabetic recovery. Likewise, a group of 15 patients (5.9%) was phenotypically similar to the group whose diabetes remained unresolved postoperatively, but had a genetic profile pre-empting them to experience remission.

Although including genomic information increased classification performance overall, a few patients (*n*=6 and 8, Figure 3a) were incorrectly classified. These patients might have been clinically misclassified or been subjected to diabetes remission mechanisms emerging over a longer period of time. Remission end point and diabetes definitions are other limitations of the study. Diabetes remission is here defined as a discontinuation of antidiabetic treatment after 30 days. It would be interesting to see how the models proposed here perform over alternative definitions of diabetes remission.

A number of studies^9,16–18,28^ have recently been conducted with the aim of elucidating clinical traits associated with diabetes remission following bariatric surgery — often with reasonable performance. However, performances are reported differently across studies and are hard to directly compare. To our knowledge, this is the first study that reduces patient similarity across the cohort, and a rigorous cross-validated performance is reported, which should provide higher generalisability in other cohorts of the selected models and their performance. Previous studies have shown associations of higher C-peptide concentration and shorter duration of diabetes with diabetes remission.^16,29^ These factors, as are often related to diabetes severity, are likely to improve the remission predictions had they been measured in the present data set. Our study nevertheless highlights insulin treatment, insulin-sensitising agents, baseline HbA1c and baseline serum insulin as important clinical features, and also points to a higher likelihood of diabetes remission for patients with a less severe diabetes.

Interestingly, several of the prioritised genes are known to have a role in insulin secretion, insulin sensitivity or obesity. The ATP-Binding Cassette, Sub-Family A, Member 1 (*ABCA1*) is a cholesterol efflux pump regulating cellular cholesterol. Studies suggest a possible relationship between ABCA1, beta-cell cholesterol homeostasis and insulin secretion, although the precise mechanism remains unresolved. In mice, absence of pancreatic islet ABCA1 seems to cause intracellular cholesterol accumulation and beta-cell dysfunction and, at some level, affected insulin secretion.^30–33^ A human study further suggests the importance of ABCA1 for normal function of the beta-cell where loss-of-function heterozygous carriers showed impaired insulin secretion without insulin resistance,^34^ although the precise role of ABCA1 mutations on pancreatic beta-cell function is not universally agreed on,^35,36^ perhaps pointing at subgroup effects where further context-dependent studies or analyses are needed. Different steps in the insulin secretion pathway might be affected by cholesterol overload; suggested pathways include regulation of glucokinase, a key factor in beta-cell glucose metabolism, via nNOS.^30^ Furthermore, hepatic expression of ABCA1 has been shown to improve glucose tolerance in mice.^37^ In macrophages, hepatic and intestinal tissue expression of ABCA1 can be regulated by the bile acid nuclear receptor Farnesoid-X-Receptor and the oxysterol nuclear receptor Liver-X-receptor^38^, where bile acids are known to increase post surgery.^39^ Both ligand-activated transcription factors are known to have important roles in the enterohepatic circulation of bile acids, the metabolism of lipids and glucose and—more interestingly—the pathogenesis of type 2 diabetes.^40,41^ Furthermore, mouse studies have shown that a functional Farnesoid-X-Receptor pathway is important for beneficial effects of bariatric surgery such as weight loss and improved glucose tolerance.^39^ Tomosyn-2 (*STXBP5L*) inhibits insulin secretion from the pancreatic beta cells.^42,43^ More specifically, it inhibits the formation of the SNARE complex that is central to the fusion of insulin granules with the plasma membrane and consequent release of insulin into the bloodstream.^44^ Several insulin secretagogues have, furthermore, been shown to induce phosphorylation and consequently degradation and/or inactivation of tomosyn-2.^44^ Prokineticin-2 (*PROK2,*) is an anorexigenic peptide hormone, which binds to two similar G protein-coupled receptors (PKR1 and PKR2). Signalling from PKR1 has several beneficial effects, including promoting peripheral transcapillary insulin uptake and hereby sensitising the peripheral organs to insulin, decreasing food intake by central appetite regulation and preventing adipose tissue expansion by inhibiting pre-adipocyte proliferation and differentiation into adipocytes.^45^ The three SNPs corresponding to these genes were shown to be in opposition to the clinical background in the prediction models (Figure 2d), and it would be very useful to monitor these SNPs in other cohorts.

We have in the present study proposed and applied a machine-learning-based approach for ranking clinical and genomic features, allowing nonlinear combinations, in order to uncover factors at play in diabetes remission triggered by bariatric surgery. However, the general framework holds the potential to integrate additional data types, such as environmental factors or metabolite concentrations on an equal footing. We propose that the combination of contextual information and genomic information holds the key to uncover more biological findings than genomics can accomplish alone and *vice versa*, that the use of clinical information can be better informed by including certain genomic markers. This is of particular interest in selecting patients for bariatric surgery. For instance, there is interest in future studies determining predisposing factors towards diabetic remission in low-BMI patients. Mechanistic insights derived from multivariate models will lead to improved understanding of the continuum of diabetic remission response after surgery in different patient subgroups.

Many GWAS data sets are underpowered for using strict statistical methods; hence, we hope that the use of heuristic approaches, as outlined here, can be useful in mining existing data sets, and proposing actionable hypotheses. Indeed, it is noteworthy that we, by such an approach, could identify a set of predictive SNPs even though none of the SNPs were significant at the 0.05 level after correcting for multiple testing in the GWAS. Although such studies do not have the power of classical statistical approaches, they do offer a paradigm for working with limited sample sets in identifying prognostic factors and generating testable and clinically understandable hypotheses.

## Materials and methods

### Data

Data used in this study originated from the Geisinger eMERGE Genome-Wide Association Studies of Obesity, where 982 primarily Caucasian, extremely obese patients had undergone a Roux-en-Y gastric bypass surgery. The data set was obtained through the dbGaP (study accession phs000380.v1.p1).^46^ A subset of the cohort (*n*=460, but three were excluded because of high rate of missing genotypes) was on diabetes medications (biguanides, insulin, sulfonylureas and insulin-sensitising agents) before surgery. For these patients, diabetes remission was defined as discontinued used of diabetes medication within 30 days after surgery (317 remitters and 140 nonremitters). The data set included 15 pre-surgery clinical covariates (presented in Table 1; height was excluded because of multicollinearity with weight and BMI, and tobacco use was excluded because of a high number of missing observations (>28%)).

Genotyping was performed with the CardioMetaboChip (Illumina, San Diego, CA, USA) array, designed for genotyping SNPs associated with metabolic and cardiovascular diseases and traits, with available genotype data for 86,444 SNPs. SNP and gene annotations were taken from the CardioMetaboChip Gene Annotation file with map positions in build 36 coordinates.

Data processing, statistical analysis and machine-learning were performed in the R statistical software, and single-locus associations with PLINK v1.07 (https://www.r-project.org and http://pngu.mgh.harvard.edu/purcell/plink/; ref. 47) as described below. A flowchart detailing the workflow with references to the corresponding figures and tables is depicted in Supplementary Figure S2.

### Generating an enriched subset of candidate SNPs by utilising prior biological knowledge

Single SNP allelic associations with diabetes remission were tested with logistic regression under a multiplicative model of associations (Figure 1b), adjusted for sex and age and with standard quality-control filters applied (exclude SNPs with minor allele frequency <5%, deviation from Hardy–Weinberg equilibrium (*P*<0.0001) or missingness rate >10% and patients with missing genotype rate >10%). No sign of population stratification was detected; the genomic inflation factor (*λ*) was 1.0, and there was no sign of inflation of the associated *P* values in the qq plot of observed versus expected −log_10_(*P* value) (Supplementary Figure S3).

The top 200 SNPs with the lowest *P* values (3.54×10^−6^–1.96×10^−3^) were used as seeds for the subsequent analysis (Figure 1c). The set of seed SNPs was expanded in a biologically relevant context by including SNPs associated with protein–protein interaction partners for gene products with an associated top 200 SNP (Figure 1d). Protein–protein interaction partners were retrieved from InWeb5.5, a high-confidence human protein–protein interaction network created from experimental data from both human and model organisms^48^ that has recently been updated (unpublished) and covers 14,536 proteins with 337,951 interactions. Finally, SNPs were removed if they were in linkage disequilibrium (*r*^*2*^>0.8, keeping the SNP with lowest *P* value) or with *P* values >0.2, resulting in 960 SNPs (Figure 1e).

### Reducing patient similarity

Typically, there are two pitfalls in estimating performance; one relates to the cross-validation set-up where one is in danger of overfitting the data, which we address through a rigorous approach as outlined in Supplementary Figure S4, and the second challenge relates to high similarity of the patients. Data similarities in the training and test sets will lead the algorithm into learning to reproduce its own input rather than being able to interpolate and extrapolate sufficiently. Thus, a non-redundant data set was generated by removing phenotypically similar patients using a modified version of algorithm 2 of Hobohm *et al.,*^49^ which favours removing similar patients with many missing observations, resulting in a more complete final data set. Similar patients were defined by having a Gower similarity coefficient^50^ of phenotype vectors above 0.925, as the data set contains both metric and dichotomous variables, which resulted in a final data set of 268 individuals (154 remitters and 114 nonremitters; Supplementary Figure S5). This patient similarity-reduced data set of 268 individuals was used in neural network models outlined below.

### Ranking of features, network training and validation

For network training and testing, a standard feed-forward-back-propagation network using one hidden layer with three units was applied using the nnet^51^ and caret^52^ R-packages. This artificial neural network implementation ignores individuals with missing information; therefore, only the subset of the 268 individuals with complete information for the included features was used. Regularisation with a weight decay parameter of 1 was included to minimise risk of overtraining the rather small data set. Training of the weights in the neural network was performed with a maximum of 1,000 iterations and otherwise default parameters using training data. To improve network training, dichotomous variables were encoded as 0.05 and 0.95, continuous variables were log-transformed and SNP data were additively encoded as one-column vectors with counts of minor alleles ({0,1,2}). Continuous variables were further standardised within the cross-validation, using the mean and s.d. for the given train data-split for standardising both the train and test data set (Supplementary Figure S4). In summary, we implemented a standard artificial neural network approach using good practices and building on experience in the use of artificial neural networks in biological context.

Feature selection was performed by a sequential forward feature selection approach within a nested cross-validation set-up (see Supplementary Figure S4 for a schematic representation), with five outer folds and five inner folds, where the inner split was repeated five times; in total, 125 sets of features selected (this feature selection scheme represents over half a million tested models). Subjects without diabetes remission were equally distributed across the different cross-validation splits. AUC for test performance was employed as performance measure for selecting features. In cases of equally good features, one was randomly selected in the feature selection approach, and features were added as long as AUC improved by at least 0.01. This procedure was first applied to clinical features. Second, fixed sets of top-ranked clinical features constituted the basis for evaluating individual SNP importance. This approach appeared to be more successful in workflow complexity than considering the clinical and SNP features simultaneously. The final set of features was determined by all features, which was selected at least once in each of the five outer cross-validation folds, and at least *X*-times over all 125 feature selections, where *X* is 45 for clinical features and five for SNPs. The first condition is an attempt to reduce the potential risk of circularity in features selected and internal validation.

Relative importance of input variables was made based on the code described in https://beckmw.wordpress.com.

For the downstream patient stratification, an artificial neural network output score of 0.5 was used to classify predicted nonremitters from remitters. Performance improvements, as reported by categorical and continuous NRI and integrated discrimination improvement, were calculated using the PredictABEL R-package.

## Figures and Tables

**Table 1:** Baseline patient characteristics associated with diabetes resolution for the nonredundant subset of 268 patients from the eMERGE cohort

*Variable*	*No diabetes resolution*	*Diabetes resolution*	P
No. of patients	114	154	
Male sex	39 (34.2%)	44 (28.6%)	0.393
**Age at time of bariatric surgery** (years)	**52.0 [45.0;59.0]**	**48.0 [38.0;56.8]**	**0.004**
Weight before bariatric surgery (pounds)	308 [268;337]	308 [260;374]	0.423
BMI before bariatric surgery (kg/m^2^)	48.0 [43.7;55.0]	50.5 [43.4;57.8]	0.321
Alcohol use before bariatric surgery (*n*=237)	25 (25.0%)	52 (38.0%)	0.05
Tobacco use before bariatric surgery (*n*=192)	27 (33.3%)	37 (33.3%)	1
Systolic blood pressure before bariatric surgery (mm Hg)	136 [122;153]	134 [122;152]	0.649
Diastolic blood pressure before bariatric surgery (mm Hg)	74.0 [67.0;85.8]	77.0 [68.0;86.0]	0.452
Pulse pressure before bariatric surgery (mm Hg)	60.0 [49.2;73.8]	59.0 [48.0;68.0]	0.21
**Serum glucose before bariatric surgery(*n*=267)**	**131 [91.0;198]**	**101 [86.0;143]**	**0.01**
**Serum insulin before bariatric surgery(*n*=255)**	**17.2 [9.80;35.3]**	**23.2 [13.5;37.8]**	**0.031**
**Haemoglobin A1c before bariatric surgery (*n*=264)**	**8.10 [7.20;9.40]**	**6.70 [6.05;7.80]**	**<0.001**
**Use of biguanides before bariatric surgery**	**68 (59.6%)**	**127 (82.5%)**	**<0.001**
**Use of insulin before bariatric surgery**	**84 (73.7%)**	**35 (22.7%)**	**<0.001**
Use of sulfonylureas before bariatric surgery	39 (34.2%)	58 (37.7%)	0.651
**Use of insulin-sensitising agents before bariatric surgery**	**57 (50.0%)**	**51 (33.1%)**	**0.008**

Abbreviation: BMI, body mass index.

Values show the median [1st; 3rd quartiles] or number of patients and percentages (%). *P* values are shown for *χ*^2^-test (categorical variables) and Kruskal–Wallis test (continuous variables). Rows with *P* values <0.05 are shown in bold. If not otherwise stated, *n*=268.

**Table 2:** Description of the eight highest ranked SNPs by the present study, ordered according to Figure 2c

*SNP ID*	*Chr*	*Coordinate*	*Gene location*	*Gene symbol*	*Gene name*	*Regulome DB score*	*Minor allele*	*Major allele*	*MAF*	*Remission* P value	Waist-hip ratio adjusted for BMI P value
rs1688686	3	122144630	Intron	*STXBP5L*	Syntaxin-binding protein 5-like (Tomosyn-2)	6	A	G	0.280	1.75×10^−4^	8.40×10-^1^
rs9310221	3	71932386	Intergenic	*PROK2*|*RYBP*	Prokineticin-2|RING1 and YY1-binding protein	7	A	G	0.426	4.79×10^−4^	3.20×10^−1^
rs2279400	12	52867581	Intron	*SMUG1*	Single-strand-selective monofunctional uracil-DNA glycosylase 1	7	G	A	0.450	3.54×10^−6^	4.60×10^−1^
rs11600200	11	119839890	Intron	*ARHGEF12*	Rho guanine nucleotide exchange factor (GEF) 12	6	C	A	0.232	9.61×10^−4^	2.90×10^−4^
rs2190513^a^	7	42175859	Intron	*GLI3*	GLI family zinc finger 3	5	G	A	0.418	4.38×10^−3^	2.00×10^−1^
rs10230715^a^	7	42155381	Intron	*GLI3*	GLI family zinc finger 3	5	G	A	0.471	2.21×10^−3^	1.50×10^−1^
rs10820743^a^	9	106711480	Intron	*ABCA1*	ATP-binding cassette, sub-family A (ABC1), member 1	6	G	A	0.292	7.54×10^−2^	7.00×10^−1^
rs6512714	20	35874753	Intron	*CTNNBL1*	Catenin, beta like 1	6	A	C	0.356	9.35×10^−4^	9.30×10^−1^

Abbreviations: AUC, area under the curve; BMI, body mass index; Chr, chromosome; MAF, minor allele frequencies; OGTT, oral glucose tolerance test; SNP, single-nucleotide polymorphism; TF, transcription factor.

MetaboChip SNP IDs, chromosome locations and gene locations are from the MetaboChip annotation file in build 36 coordinates. RegulomeDBscore was used to identify DNA features and regulatory elements overlapping the SNP coordinates (7=none; 6=other; 5=TF binding or DNase peak). MAFs are for all the 457 participants from the cohort. *P* values are listed for the 1 out of 18 tested GIANT, DIAGRAM and MAGIC consortium studies with any nominal *P* value<0.01 (BMI^19^ waist–hip ratio adjusted for BMI;^20^ Type 2 diabetes;^21^ corrected insulin response, corrected insulin response adjusted for insulin-sensitivity index, ratio of the AUC for AUC insulin/AUC glucose, insulin-sensitivity index, disposition index, insulin at 30 min, incremental insulin at 30 min, insulin response to glucose during the first 30 min adjusted for BMI, and AUC of insulin levels during OGTT;^22^ 2 h glucose, fasting glucose, fasting insulin, and fasting insulin adjusted for BMI profiled with the Metabochip;^23^ fasting glucose and fasting insulin^24^).

a SNPs included from the protein–protein interaction network expansion.

**Table 3:** Internal validation performance for the two models: the four clinical traits alone or in combination with the eight SNPs

*Model*	*Included individuals*					*AUC mean (s.d.) for 1,000 splits on the individuals held out because of their redundant properties*
	*Same splits as used for feature selection*				*AUC mean (s.d.) for 1,000 splits*	
	*AUC*	*Accuracy*	*Specificity*	*Sensitivity*		
Clinical traits alone	0.810	0.735	0.629	0.811	0.807 (0.0054)	0.992 (0.00008927)
Clinical traits+eight SNPs	0.921	0.838	0.790	0.872	0.919 (0.0046)	0.917 (0.001072)

Abbreviations: AUC, area under the curve; SNP, single-nucleotide polymorphism.

The first four columns show internal validation and performance measures for the cross-validation splits used for feature selection and the 268 individuals remaining after excluding similar patients (as reported throughout the paper). The next column shows internal validation again based on the 268 individuals, but for 1,000 different cross-validation splits. The last column shows the AUC for the 189 individuals initially held out because of their redundant properties in terms of clinical traits. In this last column, the models are trained on the 268 included individuals but evaluated on the 189 held out individuals.
