# Supplementary material for: Ranking factors involved in diabetes remission after bariatric surgery using machine-learning integrating clinical and genomic biomarkers
Source: NPJ Genom Med. 2016 Oct 26;1:16035–. doi: 10.1038/npjgenmed.2016.35 (PMC5685313; doi:10.1038/npjgenmed.2016.35)
Supplement: Supplementary Information [file npjgenmed201635-s1.pdf]

Supplementary figure legends

Supplementary Figure S1

Manhattan plot highlighting the top 200 seed SNPs (red), the 960 expanded set enriched for biological importance (yellow) and the final eight selected SNPs (blue).

Supplementary Figure S2

Overview of the applied workflow. Dark grey boxes depict analysis steps whereas light grey boxes refer to figures and/or tables showing the corresponding results or further details of the analysis. ANN: Artificial neural network.

Supplementary Figure S3

A qq-plot showing the observed versus the expected distribution of $-\log_{10}(P\text{-values})$ for association with diabetes remission from logistic regression adjusted for sex and age using a multiplicative model of association (n = 467).

Supplementary Figure S4

Approach for forward feature selection and subsequent internal validation. (**a**) shows a schematic representation and (**b**) algorithmic details of the nested cross-validation splits of the data used for selecting clinical traits and SNPs and subsequent estimating internal model performance.

Supplementary Figure S5

(**a**) Illustration of unusual patients. Two different classes of patients are depicted in a 2-dimensional space of patient similarity, for example those who would benefit – or not – from a given treatment. In real settings, the number of dimensions of interest will vary by study and data collection breadth and quality. For a prediction algorithm to have any clinical relevance it must be able to correctly predict the unusual patients, i.e. patients whom the doctor cannot easily classify based on prior experience and medical knowledge because they have a different outcome than their otherwise mostly similar

peers (patient a) and preferable also those who are very different form the majority of the patients (patient b). (**b**) Number of retained patients after applying the modified Hobohm 2 algorithm given different Gower similarity thresholds. The vertical grey line indicated the applied 0.925 cutoff. As a further benefit, the redundancy reduction resulted in a final dataset with higher class-balance. (**c-d**) Similarity of patients before (**c**) and after (**d**) applying the modified Hobohm 2 algorithm to reduce redundancy of the dataset. The patients are clustered with Ward's hierarchical clustering using a Euclidean similarity measure. The full cohort of 467 patients contained a large cluster of phenotypically very similar patients where the vast majority also experienced diabetes remission. This cluster is substantially reduced after applying the modified Hobohm 2 algorithm, illustrating the usefulness of this redundancy reducing approach.

Supplementary tables

Supplementary Table S1

Performance improvement when adding the eight SNPs to the simpler model only containing the four clinical traits. Performance improvement is reported by categorical and continuous net reclassification improvement (NRI) and integrated discrimination improvement (IDI). The first columns show internal validation performance improvements for the cross-validation splits used for feature selection and the 268 individuals remaining after excluding similar patients (as reported throughout the paper). The last column shows performance improvements for internal validation again based on the 268 individuals, but for 1,000 different cross-validation splits.

	Value	P	Mean (s.d.)
	Same splits as used for feature selection<br>*Included individuals*		for 1,000 splits<br>*Included individuals*
NRI (categorical)	0.232	1.45×10^{-4}	0.223 (0.0244)
NRI (continuous)	1.160	2.81×10^{-29}	1.157 (0.0469)
IDI	0.230	7.33×10^{-25}	0.230 (0.0052)

Supplementary Table S2

Benchmarking of the models. Mean AUC from 1,000 repetitions of either permutation of labels or sampling of eight random SNPs (from the 960 tested SNPs, but excluding the eight selected SNPs from Figure 2c).

Model	AUC<br>Mean (s.d.)
Permute labels: Clinical traits	0.502 (0.1043)
Permute labels: Clinical traits + eight SNPs	0.503 (0.1132)
Clinical + eight random SNPs	0.814 (0.0124)
