# Supplementary figures and images for: Ranking factors involved in diabetes remission after bariatric surgery using machine-learning integrating clinical and genomic biomarkers
Source: NPJ Genom Med. 2016 Oct 26;1:16035–. doi: 10.1038/npjgenmed.2016.35 (PMC5685313; doi:10.1038/npjgenmed.2016.35)

Supplementary Figure 1

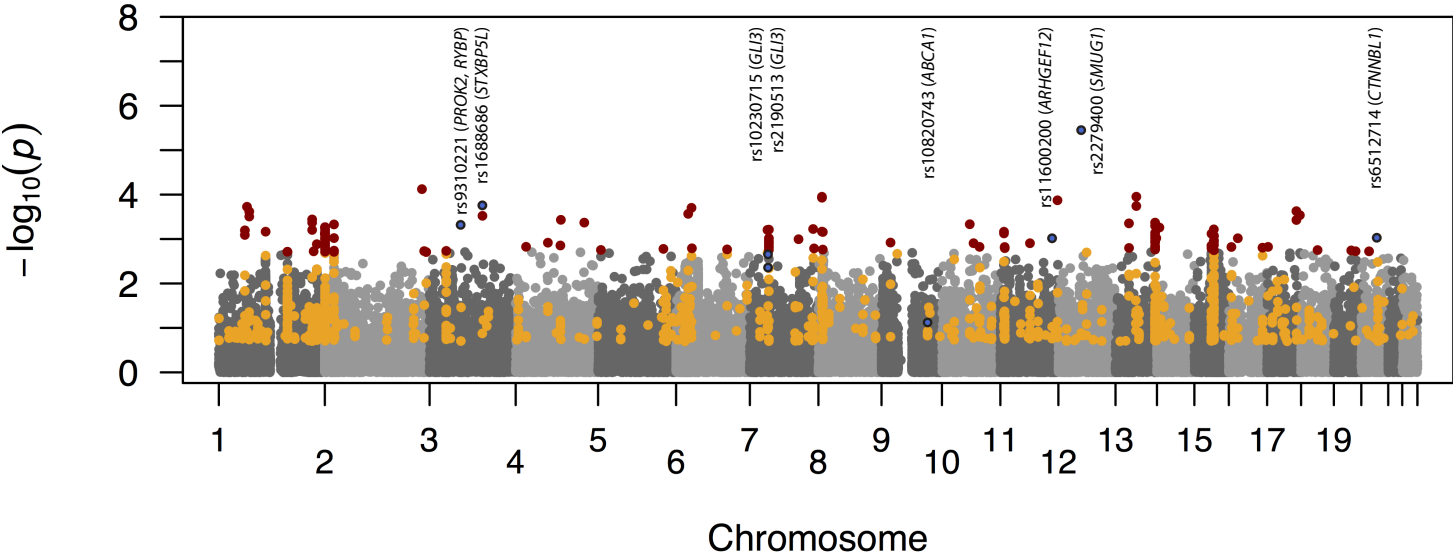

Supplement: Supplementary Figure 1 [file npjgenmed201635-s2.pdf]

Supplementary Figure 2

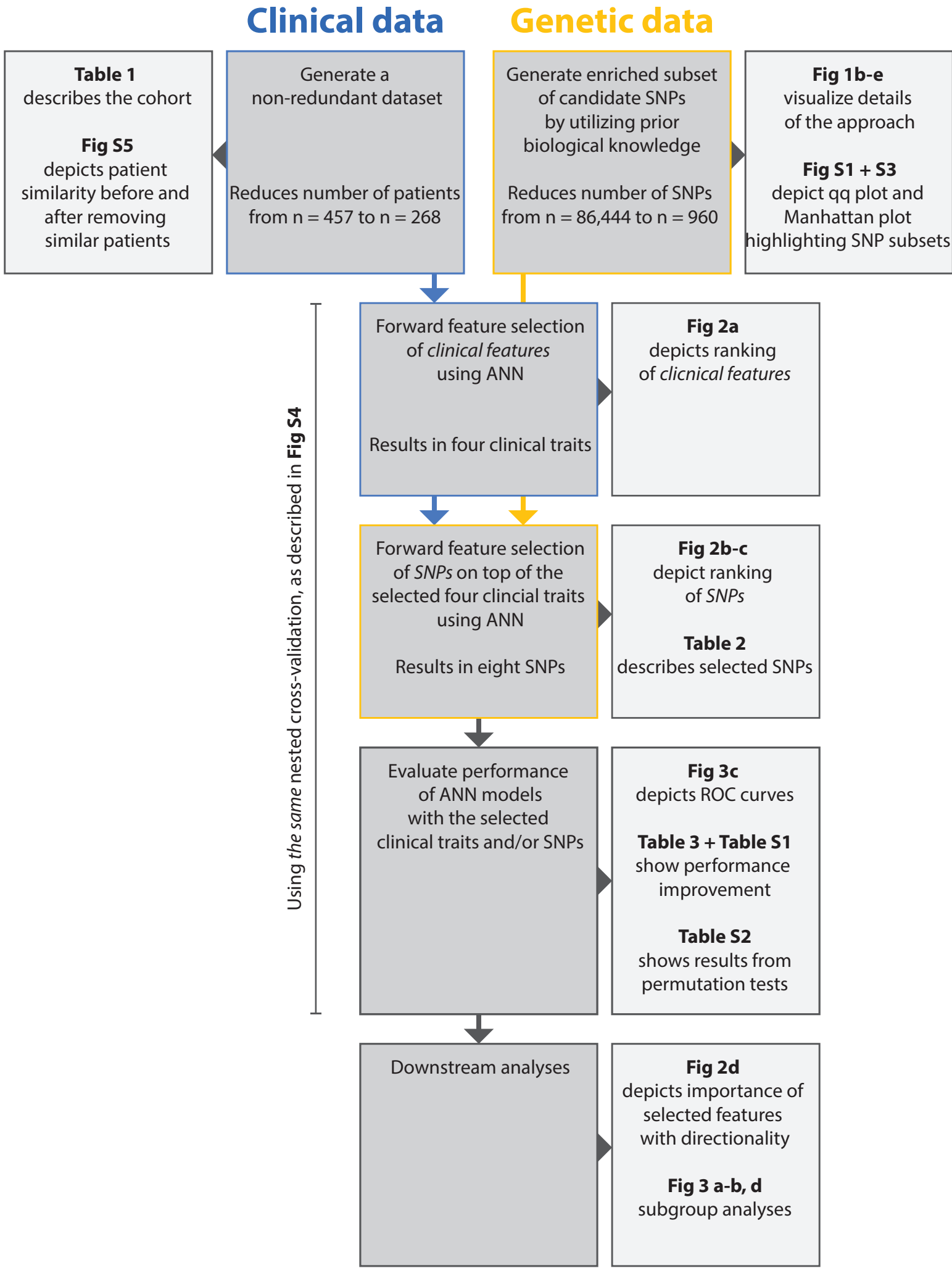

Supplement: Supplementary Figure 2 [file npjgenmed201635-s3.pdf]

Supplementary Figure 3

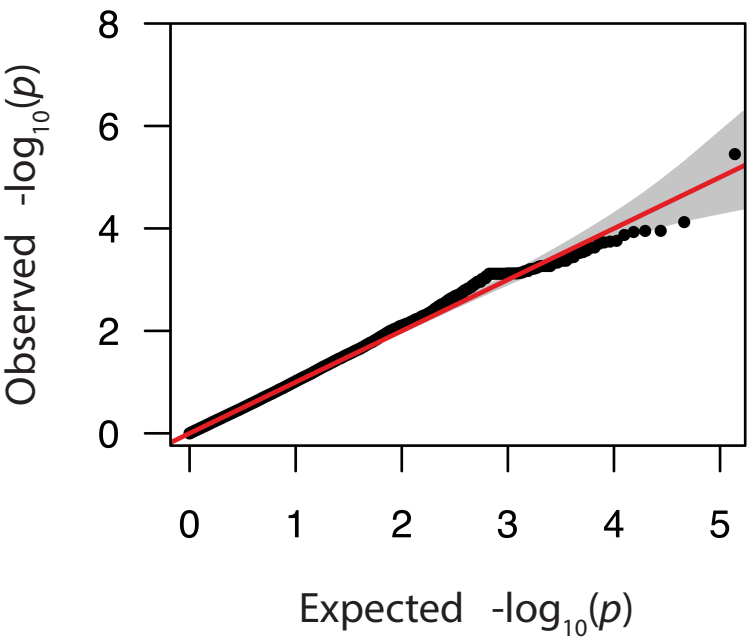

Supplement: Supplementary Figure 3 [file npjgenmed201635-s4.pdf]

# Supplementary Figure 5

a

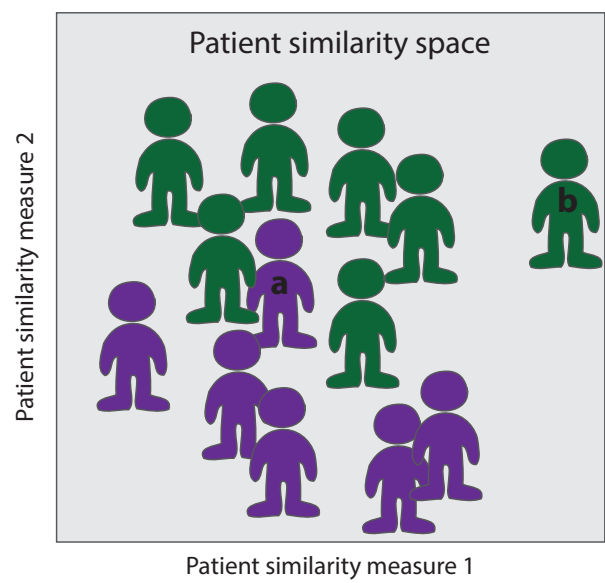

b

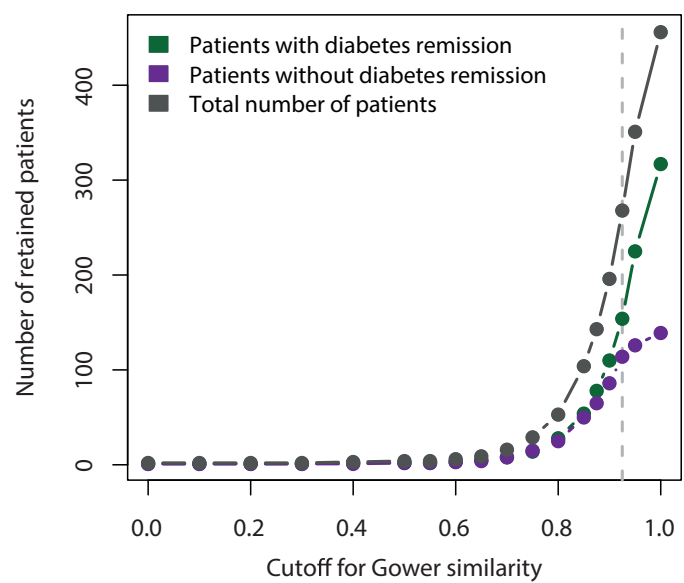

c

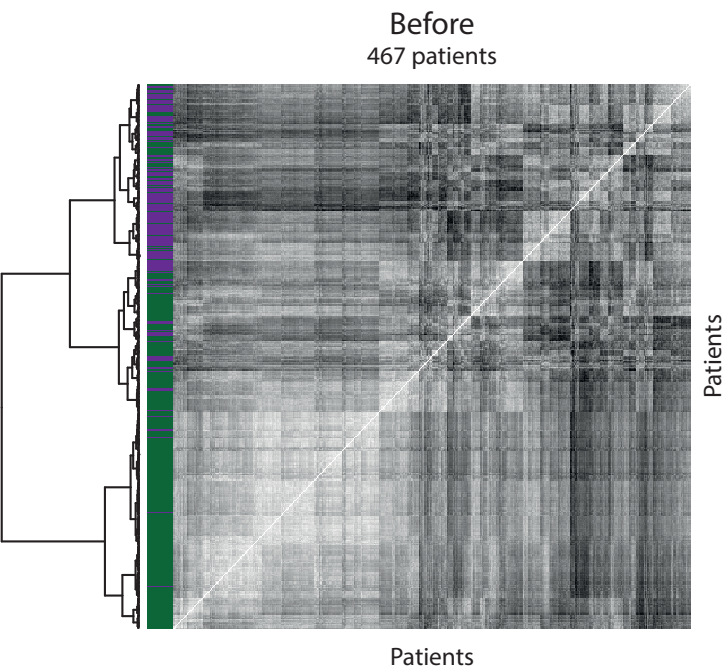

d

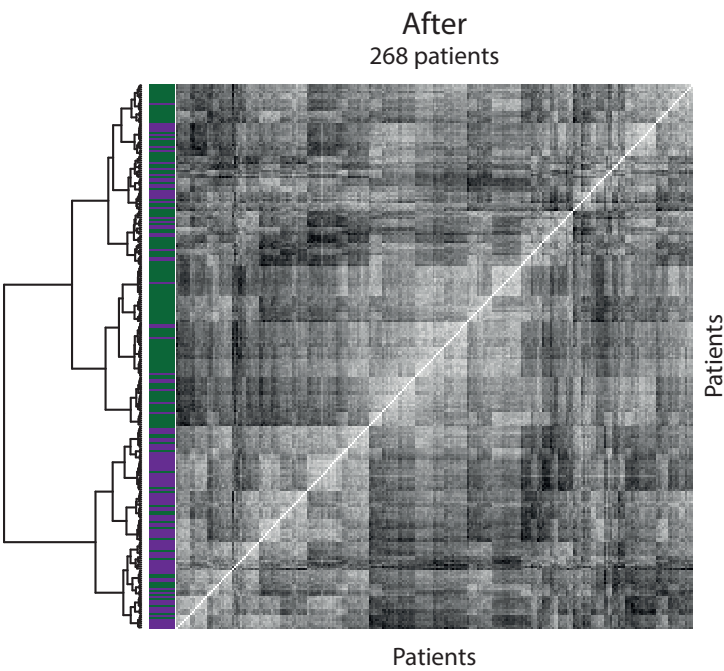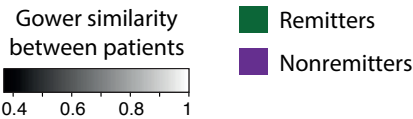

Supplement: Supplementary Figure 5 [file npjgenmed201635-s6.pdf]
