# Supplementary material for: Ranking factors involved in diabetes remission after bariatric surgery using machine-learning integrating clinical and genomic biomarkers
Source: NPJ Genom Med. 2016 Oct 26;1:16035–. doi: 10.1038/npjgenmed.2016.35 (PMC5685313; doi:10.1038/npjgenmed.2016.35)
Supplement: Supplementary Figure 4 [file npjgenmed201635-s5.pdf]

Supplementary Figure 4

a

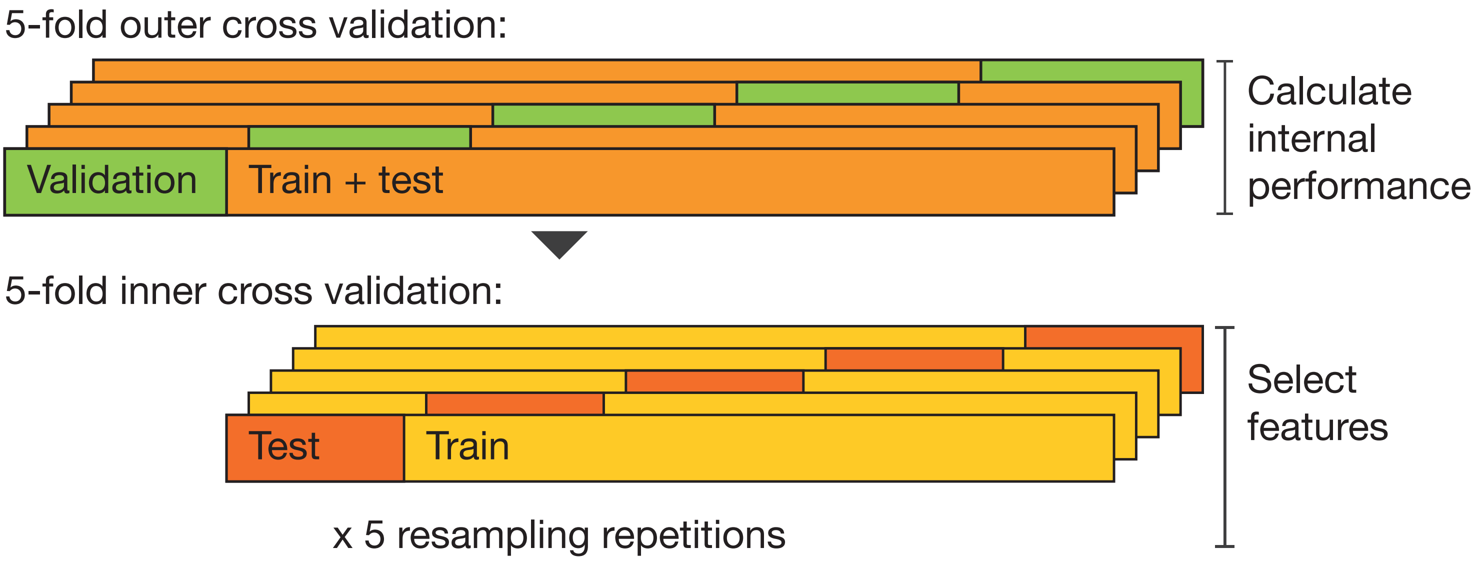

b

<u>Select features:</u>

for *each of the five outer cross-validation fold* **do**

Partition original data into **(train+test)** and **validation** sets

for *each of the five inner cross-validation fold and five resampling iteration* **do**

Partition **(train+test) data** into **train** and **test** sets

Standardise **train data**

Standardise **test data** using standard deviation and mean of **train data**

Forward feature selection by training neural network on **training data**, keep adding features as long as AUC on **test data** increase ≥ 0.01.

end

end

Determine appropriate set of features: All features selected
- at least once in each outer cross-validation fold, and
- at least *X* times over all 125 cross-validation folds/resampling iterations

<u>Calculate internal performance:</u>

for *each of the five outer cross-validation fold* **do**

Partition original data into **(train+test)** and **validation** sets

Standardise **(train+test) data**

Standardise **validation data** using standard deviation and mean of **(train+test) data**

Train neural network model using **(train+test) data** and appropriate set of features

Predict remission/non-remission for **validation** set

end

Calculate prediction performance for **validation** sets
